# Supplementary material for: Off-Hours versus Regular-Hours Implantation of Peripheral Venoarterial Extracorporeal Membrane Oxygenation in Patients with Cardiogenic Shock
Source: J Clin Med. 2023 Feb 27;12(5):1875. doi: 10.3390/jcm12051875 (PMC10003377; doi:10.3390/jcm12051875)
Supplement: Supplementary file 1 [file jcm-12-01875-s001.zip › jcm-2221587-supplementary.pdf]

Table S1. In-hospital mortality rates according to venoarterial extracorporeal membrane oxygenation indication.

	**Total**	**Regular-hours**	**Off-hours**	**p**
Acute myocardial infarction, n (%)	23 (46)	12 (48)	11 (44)	0.777
Cardiac arrest, n (%)	26 (72)	13 (68.4)	13 (76.5)	0.590
Pulmonary embolism, n (%)	12 (63.2)	6 (54.5)	6 (75)	0.361
Arrhythmic storm, n (%)	6 (37.5)	4 (33.3)	2 (50)	0.551
Acute heart failure, n (%)	5 (71.4)	4 (80)	1 (50)	0.427
Myocarditis, n (%)	3 (50)	2 (66.7)	1 (33.3)	0.414
Acute respiratory distress syndrome, n (%)	3 (75)	2 (100)	1 (50)	0.248
Takotsubo cardiomyopathy, n (%)	0 (0)	0 (0)	-	-

Table S2. In-hospital mortality rates according to the use and type of left ventricular unloading device.

	Left ventricular unloading	No left ventricular unloading	p	IABP	Impella CP	p
**Total, n (%)**	54 (51.4)	27 (65.9%)	0.115	42 (48.8)	13 (65)	0.193
**Regular-hours, n (%)**	32 (55.2)	13 (59.1)	0.752	29 (52.7)	3 (100)	0.752
**Off-hours, n (%)**	22 (46.8)	14 (73.7)	0.045	13 (41.9)	10 (58.8)	0.263

[1] IABP: intra-aortic balloon pump.

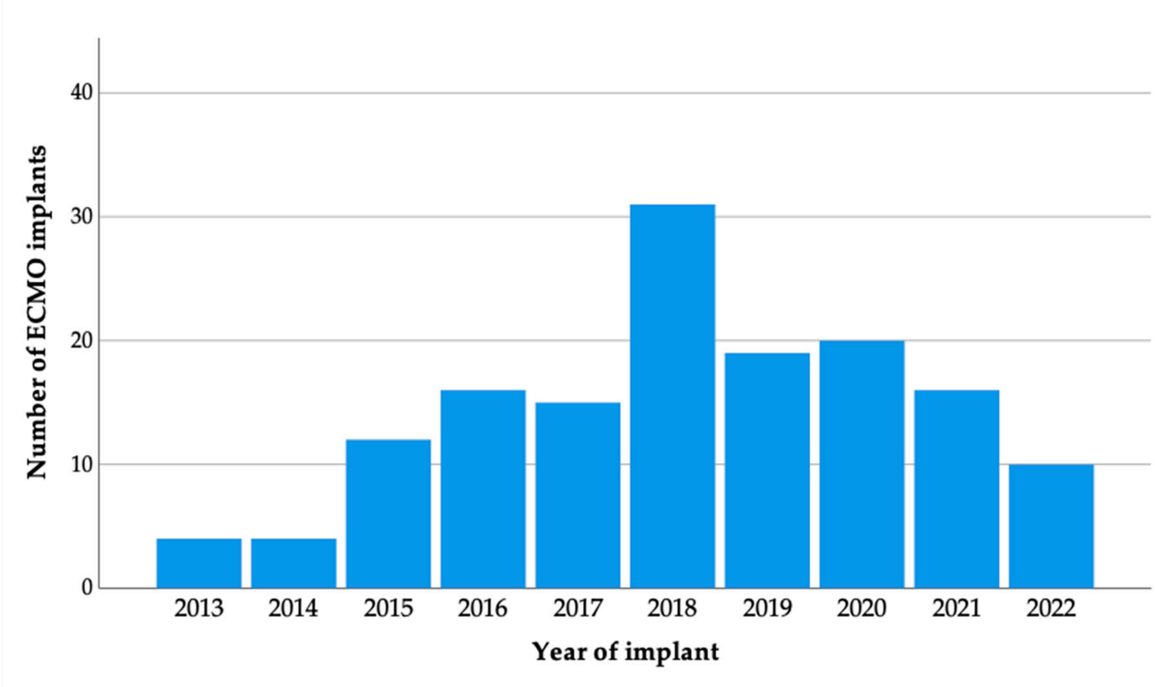

Figure S1. Number of venoarterial extracorporeal membrane oxygenation implants made each year. Please note 2022 only includes the first months.

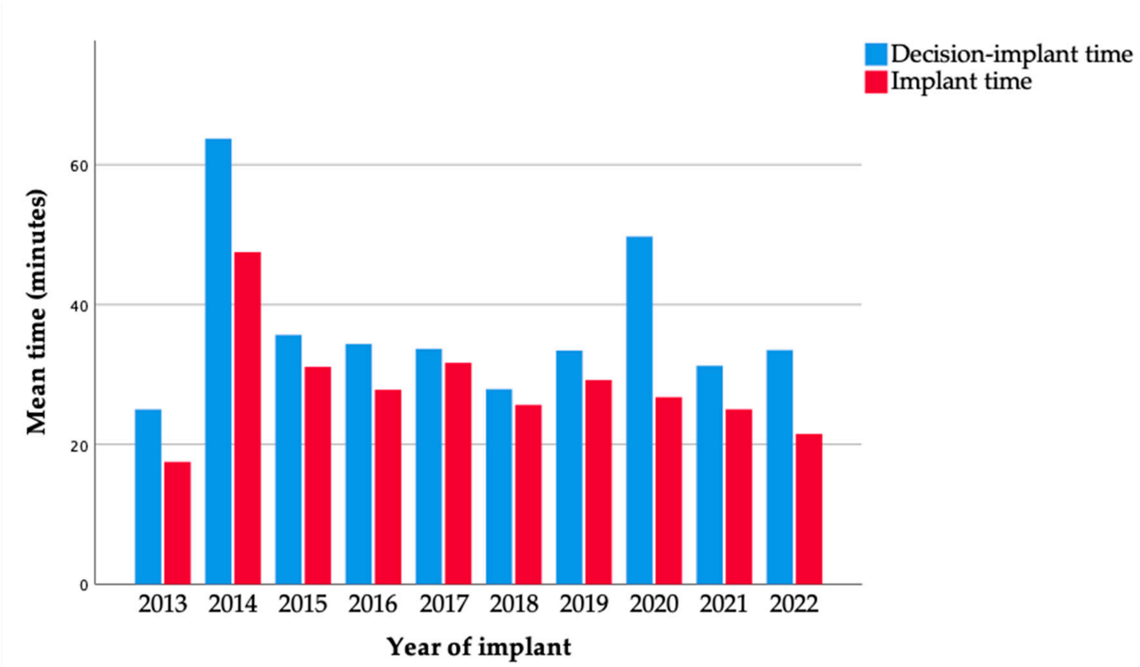

Figure S2. Implant time and decision-implant time according to year of implant. Please note year 2020 might reflect a longer decision-implant time conditioned by the effects of COVID-19 pandemic.
